# Conservative management of large mandibular odontogenic keratocysts using decompression and enucleation: a two-case report

**DOI:** 10.3389/fdmed.2026.1792569

**Published:** 2026-06-08

**Authors:** Lizet Monserrat Castillo-Real, Carlos Francisco Pérez-Sánchez, Adriana Moreno-Rodríguez, Rafael Torres-Rosas, Alfonso E. Acevedo-Mascarúa, Yobana Pérez-Cervera

**Affiliations:** 1 Departamento de Odontología, Centro de Estudios en Ciencias de la Salud y la Enfermedad, Facultad de Odontología, Universidad Autónoma “Benito Juárez” de Oaxaca (UABJO), Oaxaca, Mexico; 2 Centro de Estudios en Ciencias de la Salud y la Enfermedad, Facultad de Odontología, Universidad Autónoma Benito Juárez de Oaxaca (UABJO), Oaxaca, Mexico; 3 Laboratorio de Estudios Epidemiológicos, Clínicos, Diseños Experimentales e Investigación, Facultad de Ciencias Químicas, Universidad Autónoma “Benito Juárez” de Oaxaca, Oaxaca, Mexico

**Keywords:** conservative treatment, decompression, enucleation, mandibular lesions, odontogenic cyst, odontogenic keratocyst (OKC), recurrence

## Abstract

**Background:**

Odontogenic keratocyst (OKC) is a developmental odontogenic cyst characterized by aggressive behavior, infiltrative growth, and a high recurrence rate. Large mandibular lesions frequently involve critical anatomical structures, posing a therapeutic challenge and often leading to aggressive surgical approaches with significant morbidity. Conservative strategies aimed at reducing lesion size while preserving anatomical integrity have become increasingly relevant in clinical practice.

**Case presentation:**

This report describes two female patients, aged 22 and 55 years, presenting with large multilocular radiolucent lesions involving the mandibular body and ascending ramus. Histopathological examination confirmed the diagnosis of OKC in both cases. A conservative treatment protocol based on cystic decompression was implemented, followed by simple enucleation in one case. Clinical and radiographic follow-up demonstrated progressive reduction in the lesions and bone regeneration. In one patient, histopathological analysis after enucleation revealed replacement of the original parakeratinized epithelial lining by non-keratinized stratified squamous epithelium. Recurrence was observed in one case during follow-up, highlighting the need for prolonged monitoring.

**Discussion:**

Decompression reduced intracystic pressure, facilitated bone regeneration, and minimized the need for radical surgical intervention. This approach preserved anatomical structures and reduced treatment-associated morbidity. The observed recurrence underscores the importance of individualized treatment planning and long-term follow-up.

**Conclusion:**

Conservative management with decompression, with or without subsequent enucleation, is a viable approach for large mandibular OKCs. Careful case selection and prolonged follow-up are essential to optimize clinical outcomes and detect potential recurrence.

## 1 Introduction

Odontogenic keratocyst (OKC) is a developmental odontogenic cyst characterized by aggressive behavior, infiltrative growth, and a relatively high recurrence rate of approximately 21%, which may increase to 50% in the presence of satellite cysts (1, 2). Due to its biological behavior, including high mitotic activity of the epithelial lining and association with PTCH gene alterations, OKC is considered one of the most clinically significant odontogenic cysts (3, 4). OKC originates from remnants of the dental lamina and can occur in both the mandible and maxilla, with a predilection for the posterior mandible, particularly the molar and ramus regions (1, 5). It is more frequent in males and typically presents in the second and third decades of life, although a second peak has been described in the fifth decade (6). Radiographically, OKCs may present as unilocular or multilocular radiolucent lesions and are often associated with impacted teeth (7).

Large mandibular OKCs involving the body and ramus represent a significant surgical challenge due to their proximity to critical anatomical structures, such as the inferior alveolar nerve (8). Although multiple treatment strategies have been described, including enucleation, marsupialization, decompression, and resection, there is no consensus on the optimal approach (7, 9). Aggressive treatments may reduce recurrence but are associated with considerable morbidity (10).

Conservative approaches, such as decompression or marsupialization, have gained increasing attention as initial management strategies for large lesions. These techniques aim to reduce intracystic pressure, promote lesion shrinkage, and preserve surrounding anatomical structures (11). However, clinical evolution and histopathological changes associated with conservative management remain incompletely characterized, particularly in large mandibular lesions (12).

The present report describes the clinical, radiographic, and histopathological evolution of two large mandibular odontogenic keratocysts treated with decompression, with or without subsequent enucleation, highlighting their potential as a conservative therapeutic approach.

## 2 Case description

### 2.1 Case 1

**Figure 1:**
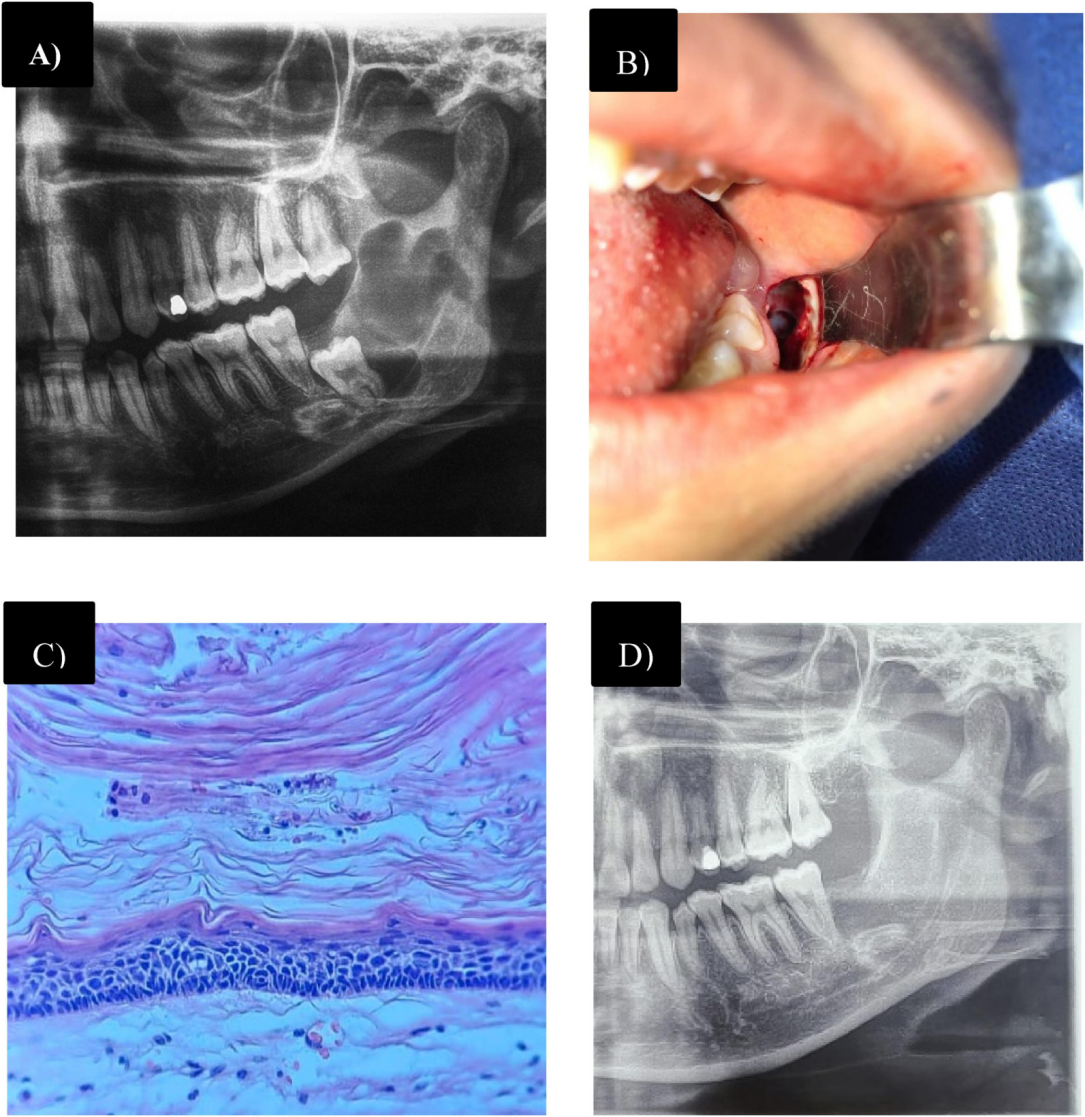
Clinical imaging and histopathology. **(A)** Multilocular radiolucent lesion involving the mandibular body, angle, and ascending ramus. **(B)** Clinically, a cystic cavity with creamy content was observed. **(C)** Microscopic examination confirmed an odontogenic keratocyst. **(D)** Bone regeneration was observed after five years of follow-up.

A 22-year-old female patient reported pain in the left lower retromolar area that was relieved by oral analgesics without clinical signs of a cystic lesion. Radiographically, a multilocular radiolucent lesion was identified in the body and ascending ramus of the left mandible (Figure 1). Based on these findings, odontogenic keratocyst, conventional ameloblastoma, and odontogenic myxoma were considered as possible diagnoses.

#### 2.1.1 Diagnostic assessment

Incisional biopsy (Figure 1B) with the extraction of the tooth involved was performed intraorally under conscious sedation and infiltration of the inferior and lingual dental nerves (2% lidocaine with 1:200,000 epinephrine). Several white, irregularly shaped, soft fragments were obtained and subjected to histopathologic examination. Histopathological examination revealed a benign cystic lesion of odontogenic origin (Figure 1C), characterized by a parakeratinized stratified flat epithelial lining, 5–8 cells thick, whose basal cells appear cylindrical and hyperchromatic, with reversed polarity, organized in a palisade and without epithelial ridges. The surface appears striated, with keratin filaments protruding towards the cystic lumen. The capsule is composed of mature fibroconnective tissue, with islands of odontogenic epithelium.

#### 2.1.2 Therapeutic intervention

Once the diagnosis was confirmed, the cyst was decompressed, the margins were extended, and a Penrose drain was placed. The patient was instructed to perform intralesional saline rinsing and oral hygiene, including 0.12% chlorhexidine rinses three times daily. Finally, antibiotics for seven days were indicated when a slight purulent discharge was found, and anti-inflammatory analgesics were also started. Clinical and radiographic follow-up appointments were scheduled every 3 months. At nine months with a significant decrease in size, it was decided to perform enucleation with adjuvant therapy. Enucleation was performed carefully due to partial adherence of the cystic lining to the surrounding bone. Additionally, a chemical curettage of the bone cavity was performed using Carnoy's solution for 5 min. Mild fibrosis of the cyst wall was observed during surgery, requiring careful dissection to achieve complete removal of the lesion while preserving the adjacent anatomical structures. The surgical specimen obtained was subjected to histopathological study, which showed re-epithelialization of the cyst area with a non-keratinized stratified squamous epithelial lining, hyperplastic in areas, no evidence of satellite cysts, and moderate chronic lymphoplasmacytic inflammatory infiltrate. Further radiographic follow-ups, scheduled every 6–9 months, showed significant lesion size reduction and lesion-free status at 5 years of follow-up (Figure 1D).

### 2.2 Case 2

A 55-year-old female patient presented to the clinic with increased volume in the left lower jaw and yellowish-white discharge in the retromolar region.

**Figure 2:**
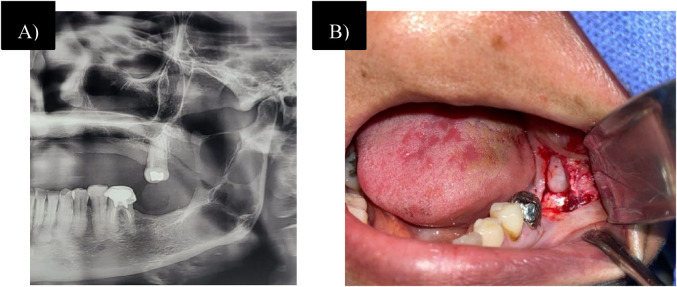
Clinical images. **(A)** Radiograph showing a multilocular lytic lesion. **(B)** A soft, whitish lesion was observed.

Radiographically, a multilocular radiolucent lesion was observed involving the retromolar area, mandibular angle, and ascending ramus, with thinning of the cortex of the anterior margin of the mandibular ascending ramus and sigmoid notch. On radiological analysis, odontogenic keratocyst, conventional ameloblastoma, and odontogenic myxoma were considered as possible diagnoses (Figure 2A).

#### 2.2.1 Diagnostic assessment

Incisional biopsy was performed intraorally under local anesthesia of the inferior and lingual dental nerves (2% lidocaine with 1:200,000 epinephrine). The incisional biopsy was subjected to histopathologic examination (Figure 2B).

**Figure 3:**
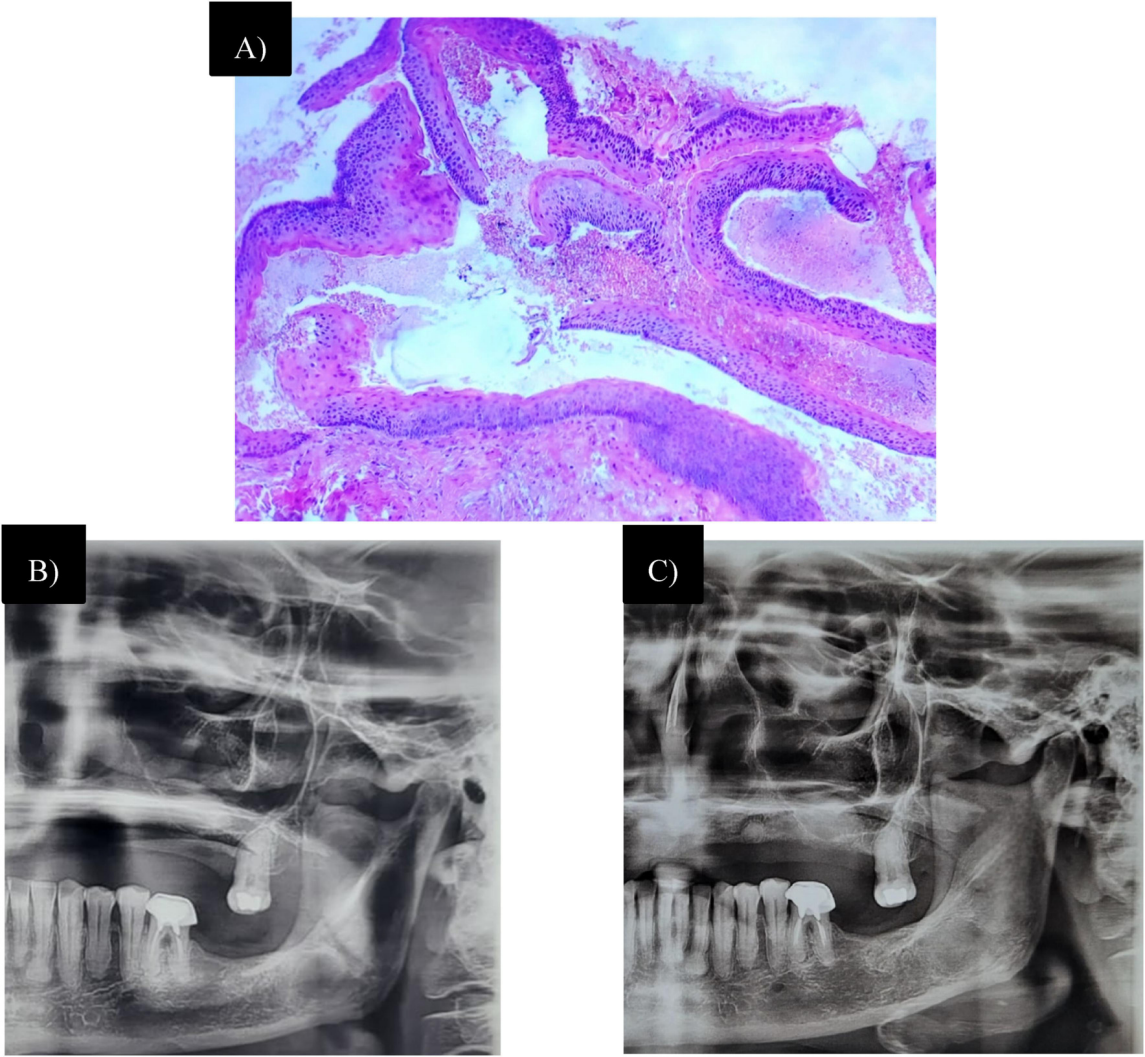
**(A)** histopathology epithelial lining showing an odontogenic keratocyst (H&E 20X). **(B)**Follow-up radiographs at 6 months. **(C)** Follow-up at 5 years with almost total bone regeneration.

Histopathologic examination (Figure 3A) revealed multiple fragments of odontogenic cystic epithelium, which shared features with odontogenic keratocysts. A parakeratinized, 6–8 cell-thick, stratified squamous epithelium with a striated surface was observed, with cuboidal basal cells that appeared palisaded, hyperchromatic, and reversed in polarity, with epithelial detachment from the underlying connective tissue. The capsule is formed by a poorly vascularized band of mature fibrous connective tissue.

#### 2.2.2 Therapeutic intervention

Once the diagnosis was confirmed, decompression with a Penrose drain and saline irrigation three times daily was indicated. A favorable response with great bone regeneration was observed at radiographic and clinical follow-up at 6 (Figure 3B). At this point, it was decided to perform simple enucleation, which was performed under local anesthesia using the same technique as the previous case. The surgical specimen obtained was subjected to histopathological study, which showed reepithelialization of the cyst without changes, no evidence of satellite cysts, and moderate lymphoplasmacytic inflammation. Periodic follow-up every 6 months was performed for 5 years, resulting in almost complete bone regeneration and limiting the need for invasive surgery. During follow-up, recurrence was detected and managed conservatively, reinforcing the need for long-term monitoring.

## 3 Discussion

Odontogenic keratocyst (OKC) remains one of the most challenging odontogenic cystic lesions because of its locally aggressive behavior and well-documented potential for recurrence (6, 13, 14). Current evidence indicates that treatment selection should be individualized based on lesion size, anatomical location, patient age, cortical involvement, and the relationship with adjacent vital structures. Large mandibular lesions extending to the angle and ramus are particularly difficult to manage because aggressive surgery may result in substantial morbidity, including loss of bone continuity, facial asymmetry, tooth loss, and injury to the inferior alveolar nerve. Contemporary multicenter and evidence synthesis studies support this individualized approach rather than a single universal treatment algorithm (13, 14).

In the present report, decompression was selected as the initial treatment in both cases because the lesions were large, multilocular, and in close proximity to important mandibular structures. In this context, decompression provides a conservative strategy to reduce intracystic pressure, promote gradual lesion shrinkage, and facilitate subsequent tissue repair while avoiding immediate radical resection (15–17).

An important contribution of the present cases is the documentation of a favourable tissue response after decompression. In Case 1, the surgical specimen obtained after secondary enucleation showed replacement of the original parakeratinized epithelial lining by non-keratinized stratified squamous epithelium with chronic inflammatory infiltrate and no evidence of satellite cysts. This finding supports the concept that decompression may induce epithelial modification and reduce the aggressiveness of the cyst lining, facilitating later removal by a more conservative procedure.

The choice of conservative treatment, however, must be interpreted cautiously. Recent higher-level evidence indicates that recurrence rates vary substantially by treatment modality and follow-up duration (16–18). An overview of systematic reviews published in 2024 concluded that recurrence may be reduced when enucleation is preceded by marsupialization or decompression; however, the methodological quality of the available evidence remains limited. In parallel, a network meta-analysis reported that adjuvant approaches after enucleation may reduce recurrence, although the certainty of evidence remains moderate (17).

In the present cases, recurrence was observed in one patient during follow-up, which underscores the intrinsic biological behaviour of OKC. Importantly, this finding should not be interpreted as a failure of conservative management, but rather as a reflection of the well-documented recurrence potential of these lesions. Therefore, long-term clinical and radiographic monitoring remains essential. This observation reinforces the need for risk-adapted treatment planning rather than a uniform surgical approach.

Another important consideration is imaging. For extensive mandibular lesions, cone-beam computed tomography would have provided a more precise three-dimensional assessment of cortical perforation, lesion volume, and proximity to the inferior alveolar nerve. In our clinical setting, CBCT was unavailable due to economic constraints, and evaluation relied on panoramic radiography and clinical examination. This limitation should be acknowledged in light of current standards of care.

The surgical aspects of conservative management also deserve emphasis. Secondary enucleation after decompression may be technically demanding due to fibrosis and partial adherence of the cystic lining to surrounding bone. In both cases, careful dissection was required to preserve adjacent structures while attempting complete removal. This should be recognized to avoid underestimating the complexity of this approach.

Overall, these findings support decompression, with subsequent enucleation with or without adjuvant therapy, as a useful conservative alternative in selected large mandibular OKCs. Their value lies not in proposing a new technique, but in documenting clinical, radiographic, and histopathological evolution under conservative management. Longer follow-up and larger comparative studies are required to better define recurrence risk and optimize treatment selection. These findings align with current evidence favoring minimally invasive strategies in anatomically complex OKCs.

### 3.1 Limitations

This report has several limitations. First, it includes only two cases, limiting the generalizability of the findings and precluding definitive conclusions about treatment efficacy. Second, cone-beam computed tomography was unavailable, and lesion assessment and follow-up relied on panoramic radiography and clinical examination, which may have underestimated lesion extent and anatomical involvement. Third, odontogenic keratocysts are characterized by late recurrence; therefore, the follow-up period can be extended beyond 5 years to draw conclusions about long-term disease control.

Additionally, as a case report, this study is inherently subject to selection bias and does not allow direct comparison between treatment modalities. Finally, interpretation of these findings should be cautious, as the existing literature on OKC management is largely based on retrospective studies with heterogeneous treatment protocols.

## 4 Conclusion

This report suggests that large mandibular odontogenic keratocysts can be effectively managed with conservative decompression, with or without adjuvant therapy, after enucleation, thereby reducing surgical morbidity while preserving adjacent anatomical structures. However, long-term clinical and radiographic follow-up remains essential because recurrence may occur several years after treatment.

## Data Availability

The original contributions presented in the study are included in the article/Supplementary Material, further inquiries can be directed to the corresponding author.
